# Island-Model Genomic Selection for Long-Term Genetic Improvement of Autogamous Crops

**DOI:** 10.1371/journal.pone.0153945

**Published:** 2016-04-26

**Authors:** Shiori Yabe, Masanori Yamasaki, Kaworu Ebana, Takeshi Hayashi, Hiroyoshi Iwata

**Affiliations:** 1 Department of Agricultural and Environmental Biology, Graduate School of Agricultural and Life Science, The University of Tokyo, Bunkyo, Tokyo, Japan; 2 Food Resources Education and Research Center, Graduate School of Agricultural Science, Kobe University, Kasai, Hyogo, Japan; 3 Genetic Resources Center, National Institute of Agrobiological Sciences, Tsukuba, Ibaraki, Japan; 4 Agricultural Research Center, National Agriculture and Food Research Organization, Kannondai, Tsukuba, Ibaraki, Japan; Michigan State University, UNITED STATES

## Abstract

Acceleration of genetic improvement of autogamous crops such as wheat and rice is necessary to increase cereal production in response to the global food crisis. Population and pedigree methods of breeding, which are based on inbred line selection, are used commonly in the genetic improvement of autogamous crops. These methods, however, produce a few novel combinations of genes in a breeding population. Recurrent selection promotes recombination among genes and produces novel combinations of genes in a breeding population, but it requires inaccurate single-plant evaluation for selection. Genomic selection (GS), which can predict genetic potential of individuals based on their marker genotype, might have high reliability of single-plant evaluation and might be effective in recurrent selection. To evaluate the efficiency of recurrent selection with GS, we conducted simulations using real marker genotype data of rice cultivars. Additionally, we introduced the concept of an “island model” inspired by evolutionary algorithms that might be useful to maintain genetic variation through the breeding process. We conducted GS simulations using real marker genotype data of rice cultivars to evaluate the efficiency of recurrent selection and the island model in an autogamous species. Results demonstrated the importance of producing novel combinations of genes through recurrent selection. An initial population derived from admixture of multiple bi-parental crosses showed larger genetic gains than a population derived from a single bi-parental cross in whole cycles, suggesting the importance of genetic variation in an initial population. The island-model GS better maintained genetic improvement in later generations than the other GS methods, suggesting that the island-model GS can utilize genetic variation in breeding and can retain alleles with small effects in the breeding population. The island-model GS will become a new breeding method that enhances the potential of genomic selection in autogamous crops, especially bringing long-term improvement.

## Introduction

Cereals account for a large share of the human food supply [1], suggesting that an increase in yield of cereals will lead to a stable world food supply and suggesting the necessity for feeding the world population, which is expected to reach nine billion by 2050 [2,3]. Most cereals, e.g. rice (*Oryza sativa* L.), wheat (*Triticum aestivum* L.), and barley (*Hordeum vulgare* L.), are autogamous plant species. In a conventional breeding program of an autogamous plant species, the size of linkage blocks remains larger than that expected for an allogamous species. That fact explains the low probability of generating novel combinations of alleles of genes in a breeding program. Bulk population methods and pedigree methods for breeding, by which breeders repeat selfing and selecting inbred lines, are used commonly for the genetic improvement of autogamous plant species [4,5]. The expected levels of breakup of initial linkage blocks through infinite repetitions of self-pollination are the same level of two to three cycles of random mating [6]. Recurrent selection, by which selection and crossing of selected individuals are performed repeatedly, was proposed to solve this issue, as suggested by Fujimaki [7]. However, in practice, it might cause different issues, one of which is inaccurate selection based on single plant evaluation. Genomic selection (GS) [8] is expected to solve the problem of recurrent selection because GS enables us to evaluate plants according to their marker genotypes on a single-plant basis. For instance, Rutkoski et al. [9] described the efficiency of recurrent selection using GS in stem rust resistance in wheat. They emphasized that the increase of recombination events facilitates the combination of favorable alleles, and that it would make greater gains than conventional bulk breeding methods would. Moreover, GS enables us to skip phenotyping at each selection cycle. It therefore enables us to implement rapid-cycle genetic improvement by accelerating generation advancement. This study evaluated the efficiency of recurrent selection using GS in autogamous plant breeding via a simulation study.

This study investigates “island-model GS” as a new breeding strategy for autogamous plants, and evaluates its potential via breeding simulations. The term “island model” was derived originally from the field of population genetics, meaning that a large population is split into multiple subpopulations and that each subpopulation receives migrants from the others. Whereas selection takes place within each subpopulation, crossbreeding occasionally occurs between subpopulations [10]. Migration among subpopulations has a tendency to counteract the dispersion of allele frequency [11]. The concept of the island model in population genetics has inspired global optimization problems in the computational science research field. In optimization problems, it is often difficult to search for the global optimum of a large number of parameters because of the existence of a number of local optima when the objective function is nonlinear and non-differentiable. Evolutionary algorithms (EAs), which are heuristic algorithms used to find a better or optimal solution, are used frequently to resolve local optima issues. In EAs, individuals are constructed based on the parameters in question, which are assumed as genes of living organisms. Relations between genotypes (i.e., sets of genes) and their fitness constitute a ‘fitness landscape’. To reach the highest fitness point (i.e., to obtain the genotype that has the best genotypic value) or at least to reach a higher fitness point (i.e., to obtain a genotype that has a better genotypic value) on the fitness landscape, selection and crossing are repeated as they are in a natural population. The concept of an island model is used in the field of EAs. In the island model of EA, individuals are split into subpopulations, and selection and crossing are repeated in each subpopulation involving migrants. Whitley et al. [12] reported that the island model of EA showed better search performance than a single population model in some cases. One reason for this efficiency is that various islands (i.e., subpopulations) maintain some degree of independence and therefore explore different regions of the parameter space. The success of the island model of EA suggests that the island model concept can be effective not only in natural selection but also in artificial selection.

The concept of the island model is expected to be efficient in plant breeding. When plant breeding, breeders select plants having better genotypic values. The genotypic value of a plant can be regarded as the fitness of the plant. Because of the conceptual and procedural similarities between plant breeding and EA, the island model is expected to be useful for plant breeding. As described above, recurrent selection might be efficient for the genetic improvement of autogamous plants. When among-cultivar diversity is large, as is often the case in autogamous crops, genetic differences among segregating families become large. When we consider segregating families as islands, the islands will maintain some degree of independence. They can be considered to explore different regions of the parameter space for “optimizing QTL genotypes.” Therefore, the concept of the island model might work effectively in the recurrent selection of autogamous breeding populations.

Some risks to consider are that the algorithms in EAs are also effective in plant breeding just because of the resemblance between EAs and plant breeding. Some differences exist between EAs and plant breeding. First, mutations occur frequently in EAs, although they remain quite limited in a breeding population because of the time scale of a breeding program. Second, EAs can simulate a large population to generate wide genetic diversity at the expense of computational time, whereas the size of a breeding population is limited in plant breeding. At the same time, labor for crossing plants in autogamous species might limits the number of plants used as parents, because crossing autogamous plants is usually laborious as a result of the necessity of avoiding selfing. Third, EAs can gain high ability through a number of selection cycles in a short time using computers, whereas plant breeding requires a long time to evaluate and select plants. Although O’Hagan et al. [13] conducted breeding simulations using some concepts in EAs, they assumed a large population size and a high mutation rate from a radiation dose. It was not realistic to implement their algorithms directly in an actual plant breeding program. It is necessary to consider the restrictions on a real plant breeding program and to evaluate the efficiency of the algorithms under the restrictions.

In this study, we conducted breeding simulations with a real marker genotype data of cultivars in Asian cultivated rice. To take advantage of existing materials and their information, we assumed the use of recombinant inbred lines (RILs) derived from crosses between the existing cultivars as a training population and as an initial breeding population. We specifically examined the following three points in the simulations: (i) the efficiency of recurrent selection in autogamous crop breeding, (ii) the suitable constituent of an initial breeding population, i.e., initial populations derived from a single bi-parental cross and derived from multiple bi-parental crosses, and (iii) the efficiency of the “island-model GS,” which is proposed first for application to plant breeding in this study. Through evaluation of these three points, we examined the potential of recurrent GS and the island-model GS in breeding of autogamous plant species.

## Materials and Methods

### Marker data and position estimation

In this study, we performed breeding simulations based on real marker data of Asian cultivated rice. A dataset consisting of the genotypes of 3,102 markers for 112 rice cultivars was used in the breeding simulations. The 112 cultivars represented the geographical and historical diversity of rice cultivars developed mainly in Japan (S1 Table). DNA of the cultivars was extracted from one typical individual plant from each cultivar using CTAB method [14], and was used further for genotyping of 3,102 markers. Among the 3,102 markers, 3,071 were single nucleotide polymorphism (SNP) markers developed from the sequence of Japanese cultivars [15,16], and 31 were simple sequence repeat (SSR) markers [17]. The SNP genotyping was done using Illumina Beads Station 500G (Illumina Inc., San Diego, USA) following the manual. The physical map positions were determined based on rice genome build 4 (IRGSP Build 4, http://rgp.dna.affrc.go.jp/IRGSP/Build4/build4.html). The positions of all markers on the linkage map were necessary to simulate between-marker recombinations that occurred in meiosis. The positions of all markers that were not located on the linkage map were estimated via a polynomial regression of the linkage map positions on the physical positions using information of the rice genetic linkage map of a F_2_ population derived from a single cross between the *japonica* variety Nipponbare and the *indica* variety Kasalath [18,19] and its updated information [20]. Imputation of missing marker genotypes was held using fastPHASE ver. 1.3 [21]. The imputation was repeated 100 times, and genotypes at each imputed locus were imputed alternatively as one of two homozygous genotypes according to the occurrence proportions of the homozygous genotypes over the 100 replications [22].

### Simulation settings

The 100 markers out of 3,102 markers were assumed as quantitative trait loci (QTL) controlling a target trait in each simulation trial. The proportion of phenotypic variance explained by each QTL (i.e., the heritability of each QTL) was set to follow the equation proposed by Lande and Thompson [23] in the population constructing of the 112 cultivars. The effective number of QTL was set as 40. The sum of heritability of all QTL was set as 0.6. The genetic variation was explained only in an additive way: no dominance and no epistatic effect influenced the trait. Genotypic values were simulated using these simulated QTL effects. Phenotypic values were calculated by adding simulated environmental deviations into those genotypic values. Phenotypic variance of population composed of the 112 cultivars was standardized to be 1.0. For each breeding procedure, 100 replications of simulation were implemented.

### Breeding schemes

First, from 112 rice cultivars, seven varieties were selected: Koshihikari, Yumeakari, Hitomebore, Hatsushimo, Hinohikari, Nanatsuboshi, and Asahinoyume. We selected these cultivars to represent the genetic diversity in the 3,102 markers of the 112 cultivars. Second, six F_1_ lines derived from six bi-parental crosses were made. Koshihikari, which was a predominant variety in Japan, was used as a common parent for the six bi-parental crosses. Starting from the six F_1_ lines, six F_6_ populations were simulated with the repeated selfing and the single seed descent (SSD) procedure. These simulated populations were used as initial populations for GS breeding and as a training population for building a GS prediction model. Each F_6_ population was constructed of 180 lines (i.e., 1,080 F_6_ lines in all).

In all GS breeding schemes, 20 cycles of GS were conducted. A prediction model was built from phenotypic values and marker genotypes of the initial populations (i.e., six F_6_ populations consisting of 1,080 lines) and was used throughout the 20 cycles. The selection intensity was set as 10%. Instead of random mating, a single-round robin [24] was used, in which crosses were conducted as a chain, i.e., plant1 × plant2, plant2 × plant3, …, plant *S* × plant1 among *S* plants, as a rule for crossing selected plants for the next generation because of the difficulty of random mating in rice (i.e., autogamous species) population. Selected plants were crossed in a round-robin design. Ten genotypes were derived from each of *S* round-robin crosses.

**Fig 1:**
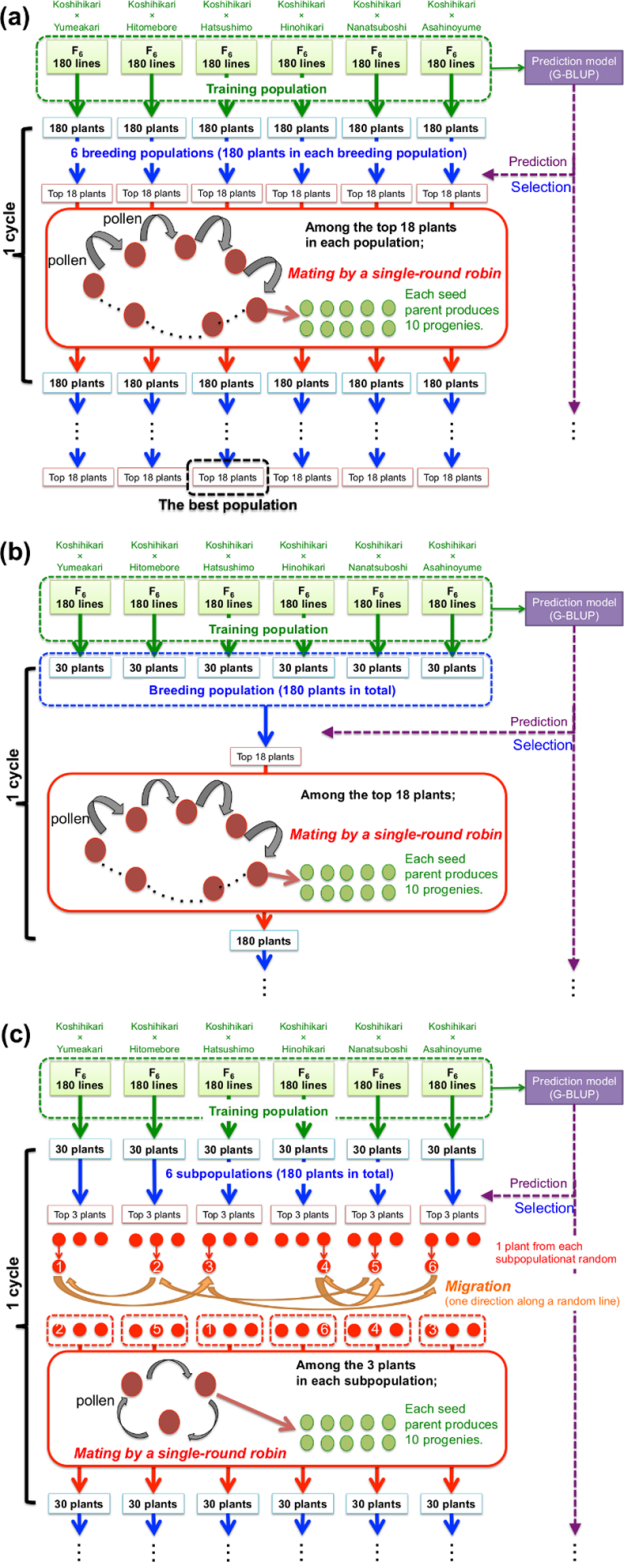
GS breeding scheme: (a) discrete GS, which improves six breeding populations each of which is derived from a single bi-parental cross; (b) bulked GS, which improves a breeding population derived from admixture of multiple bi-parental crosses; and (c) island-model GS, which improves six subpopulations that are mutually connected. The six populations are derived from different bi-parental crosses.

First, to evaluate the efficiency of recurrent selection, we compared the outcomes of GS breeding with those of RILs using the same breeding population derived from a single bi-parental cross. Second, to evaluate the impact of genetic architecture of a breeding population in GS breeding, breeding populations of two types were compared: (a) six breeding populations, each of which was derived from a single bi-parental cross (discrete GS; Fig 1A) and (b) a breeding population derived from admixture of six bi-parental crosses (bulked GS; Fig 1B). For the former, one breeding population consisted of 180 lines derived from one bi-parental cross. For the latter, six F_6_ populations derived from each of six bi-parental crosses were gathered to construct one breeding population of 180 lines, in which 30 lines came from each F_6_ population. In the discrete GS, six breeding populations of 180 genotypes, each of which was derived from a single bi-parental cross, was improved independently with GS (Fig 1A). In the bulked GS, only one breeding population, with 180 genotypes derived from admixture of multiple bi-parental crosses, was created. It experienced GS (Fig 1B). In the discrete GS and the bulked GS, the 180 genotypes were derived from the round-robin crosses of 18 plants that experienced GS. Third, to evaluate the efficiency of the island-model GS breeding, we compared the island-model GS with the bulked GS. In the bulked GS, GS was performed on a single breeding population derived from multiple bi-parental crosses (Fig 1B). In the island-model GS (Fig 1C), breeding was conducted based on six equal-sized subpopulations that were mutually connected with a small amount of migration. The initial state of each subpopulation for the island-model GS was an F_6_ population derived from a single bi-parental cross. To facilitate genetic migration between subpopulations, one of the selected plants was chosen randomly and exchanged between subpopulations every cycle. Then mating was conducted among three selected plants belonging to a single subpopulation after the migration. The migration was held in a one-directional ring design in which the six subpopulations were randomly ordered. All simulations were done using R, version 3.1 [25].

### Genomic selection model

In GS breeding, we used G-BLUP for building a model for genomic prediction. The model had the following form.

y=Xβ+Zu+ε

In that equation, ***y*** = {*y*_*i*_} is a vector of phenotypes, ***β*** is a vector of fixed effects, ***u*** is a vector of random genotypic values with *Var*[***u***] = **K***σ*_*u*_^*2*^. **K** represents the realized additive relation matrix calculated from genotypes of the 3,002 non-QTL markers with removal of monomorphic markers in the training population. **X** and **Z** respectively represent design matrices for the fixed effects and the random effects. In this simulation study, **X** was a vector of ‘1’s as the intercept of the model with the length of the number of observation; **Z** was a matrix by which an identity matrix (number of observations × number of observations) was combined with a 0 matrix (number of observation × number of prediction) by columns. **ε** is a vector of the error deviations with variance *Var*[**ε**] = **K***σ*_*e*_^*2*^. Each marker genotype is defined as 1, 0, and -1 when the numbers of the considered allele contained are respectively two, one, and zero. The R package “rrBLUP” [26] was used to build a genomic prediction model with G-BLUP.

### Summarization of results

From RILs in the initial breeding population of GS breeding, the best line (i.e., the line with the highest genotypic value) was selected. The genotypic value of the best line was used as a standard for comparing the efficiency of recurrent selection with that of breeding utilizing inbred lines.

In GS breeding, an attained genotypic value was represented as the maximum of the true genotypic values among selected plants (i.e., upper 10% of plants selected based on predicted values) at each selection cycle. Here, we assumed that a breeder can detect the best plant from the selected plants through field trials before the variety release. The average breeding values of plants belonging to a single population and the distribution of breeding values are examined to compare the attained genotypic values (i.e., the maximum of the true genotypic values among selected plants) with the population mean.

To monitor variation in selection accuracy through the breeding process, the accuracy of genomic prediction was measured by Pearson’s correlation coefficient between the predicted values and true genotypic values in each breeding cycle.

Figures show averaged values of 100 simulations in each breeding procedure. To test the significance of difference in the mean genotypic values among populations that experienced different breeding methods, we used matched-pairs Wilcoxon tests. Breeding populations in all methods were derived from an identical initial population at each replication of the simulations. The populations derived from the identical initial population were considered as a matched-pair in the Wilcoxon test.

## Results

### Genetic diversity in 112 rice cultivars

**Fig 2:**
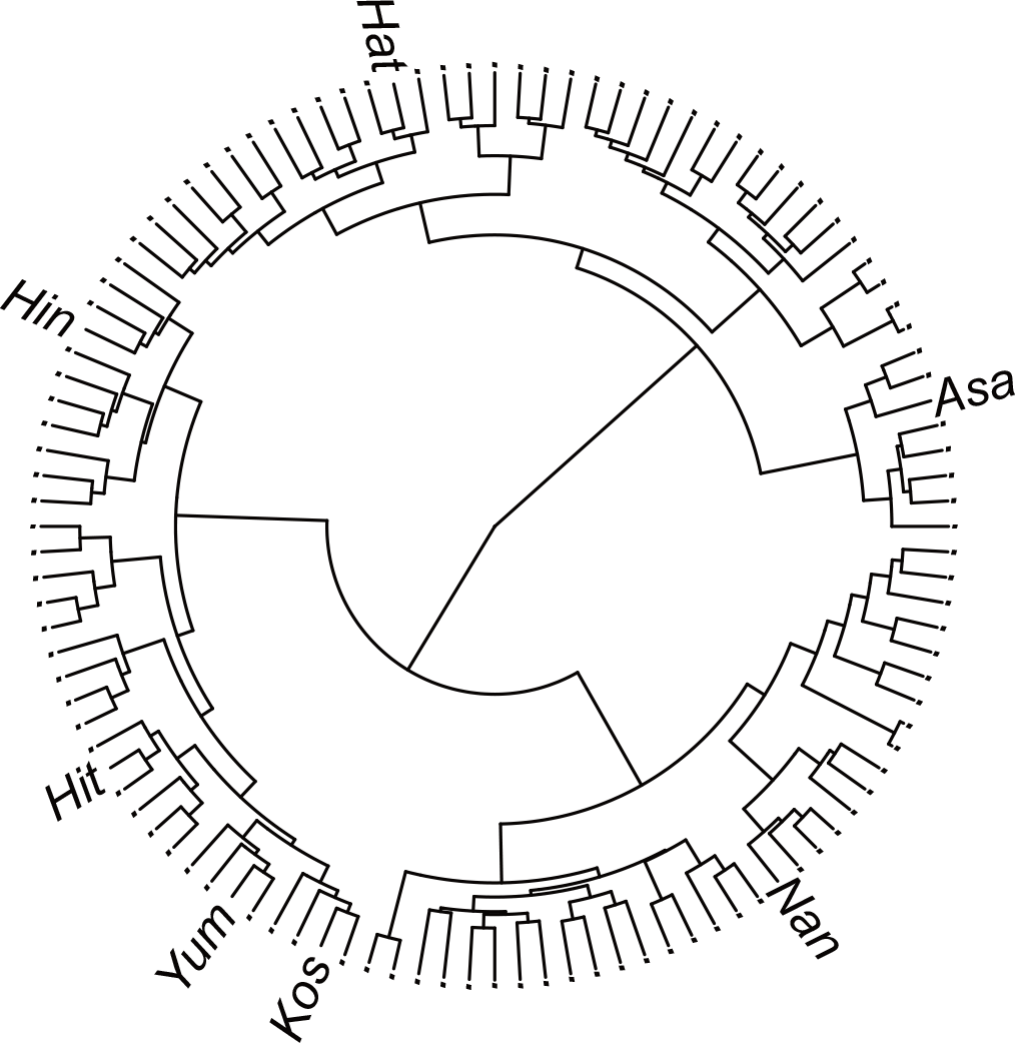
Cluster analysis of the 3,102 markers in 112 rice cultivars. Yum, Yumeakari; Hit, Hitomebore; Kos, Koshihikari; Hat, Hatsushimo; Hin, Hinohikari; Nan, Nanatsuboshi; Asa, Asahinoyume.

Fig 2 shows the result of cluster analysis of the 112 rice cultivars by 3,102 markers. The distance matrix was calculated as the Euclidean distance, in which two homozygous SNP genotypes are treated as 0 or 1. The cluster was made using Ward’s method [27]. The shared common parent in bi-parental crosses, Koshihikari, was in the bottom left of the figure. The remaining cultivars, which were used in our breeding simulation, represented the genetic diversity existing in the 112 cultivars.

### GS breeding designs

**Fig 3:**
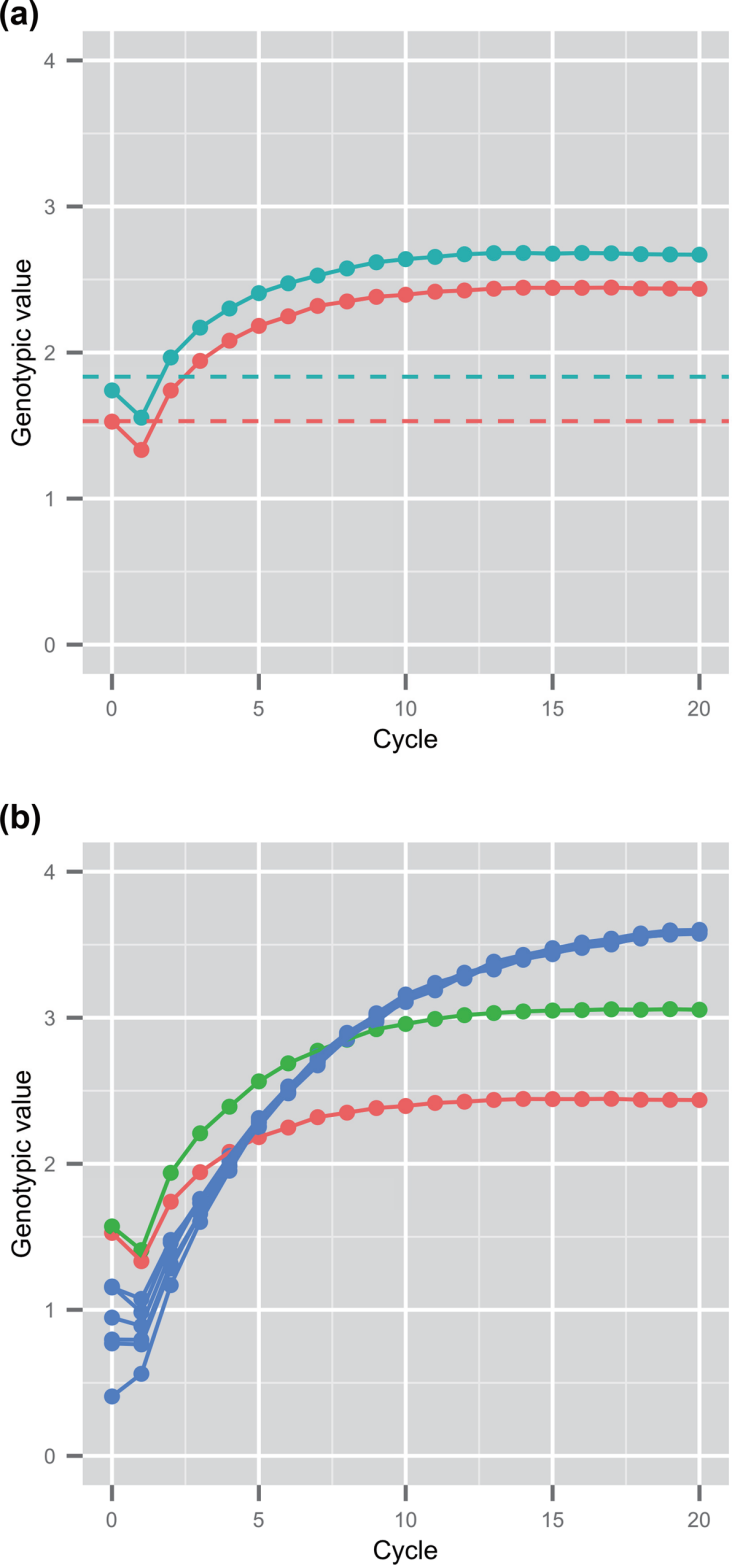
Genotypic values attained through selection cycles. Maximum of the true genotypic values among selected plants. (a) Solid lines show results of the discrete GS breeding. Dashed lines represent the maximum value of genotypic values in F_6_ population derived from a single cross. Red and blue lines respectively represent the results of Koshihikari × Hatsushimo and the results of the best population in each simulation trial. (b) Red, green, and blue lines respectively present results of the discrete GS of Koshihikari × Hatsushimo population, one of the bulked GS, and one of the island-model GS.

For the breeding of the population derived from a single cross (i.e., the discrete GS and the selection among the initial population), one population that attained the highest genetic gain was reported as the best result. Fig 3(A) shows the attained genotypic values in the discrete GS. The dashed line shows the maximum value of genotypic values in F_6_ population derived from a single cross. Red lines represent the result of Koshihikari × Hatsushimo population, which attained the highest genotypic value among the six breeding populations on average (52 out of 100 replications of simulation in GS breeding). Blue lines represent the average value of the best population in each simulation. The discrete GS attained a higher genotypic value than the maximum of the F_6_ population at every cycle after the first two cycles of selection (*p* < 0.01 in results of Koshihikari × Hatsushimo population; *p* < 0.01 in results of the best population in each simulation trial). For the result of Koshihikari × Hatsushimo population, the discrete GS exceed the maximum of F_6_ in the 85 out of 100 trials after two cycles of selection, and then attained higher genotypic value than the maximum of F_6_ in all 100 trials after six cycles of selection. The result suggests the superiority of GS breeding to the conventional methods using inbred lines. The genetic gain of discrete GS, however, reached plateau after 7–8 cycles of selection.

Fig 3(B) shows the attained genotypic values in the GS breeding of three types (i.e., the discrete GS, the bulked GS, and the island-model GS). The attained genotypic values in the discrete GS show the same values as red solid line in Fig 3(A). The bulked GS, in which the breeding population derived from multiple bi-parental crosses, attained higher genotypic values than the discrete GS using the population derived from a single bi-parental cross (*p* < 0.01 at every cycle after the first two cycles of selection). After the first two cycles of selection, the attained genotypic values of the bulked GS exceeded that of the discrete GS in 62 out of 100 simulation replications. The bulked GS showed rapid genetic improvement during the early cycles and reached plateau after 12–13 cycles.

We conducted the simulation of the island model GS only for the population derived from admixture of multiple bi-parental crosses because the population derived from admixture of multiple bi-parental crosses were expected to have higher potential than the population derived from a single bi-parental cross (Fig 3B). Blue lines in Fig 3(B) show the attained genotypic values of the island-model GS using six subpopulations. The island-model GS attained lower genotypic values than the bulked GS until the seventh cycle of selection for the averaged value over 100 trials (*p* < 0.01 at every cycle after the first 10 cycles of selection). At the sixth cycle of selection, the island-model GS exceed the bulked GS in 57 out of 100 simulation replications. In the later selection cycles, the island-model GS attained higher genotypic values than the bulked GS did. The island-model GS exceed the bulked GS in 83 out of 100 simulation replications after 12 cycles of selection. The island-model GS did not reach a plateau in the first 20 cycles. Through the 20 cycles of selection, the genetic ability of all subpopulations converged to the same level even though the initial ability was different among different subpopulations.

In the island-model GS simulation, we generally assumed that (i) the number of migration individuals was set to 1 in each subpopulation, (ii) the exchange interval was one (i.e., breeder should exchange selected individuals every cycle), and (iii) one population derived from a single bi-parental cross constructed one subpopulation. These assumptions attained better results than the other assumptions, as derived below. That is, the island-model GS simulation, in which two individuals were exchanged from each subpopulation, resulted in the similar genetic ability to the island-model GS with one individuals’ exchange (S1 Fig). The simulation with different migration intervals (i.e., exchanging event was conducted every 2–5 cycles) resulted in a lower genetic gain than the island-model GS in which individuals were exchanged every cycle (S2 Fig). The simulation based on an initial population in which lines are allocated randomly to six subpopulations represented lower gain than the simulation with the initial population separated according to their parents (S3 Fig).

In all GS breeding, decreases of the genotypic values were observed after the first selection cycle (Fig 3). S4 Fig shows two examples of simulation trials during the first three cycles of bulked GS. At the first selection cycle, the average genotypic value of the breeding population improved. The best genotypic values, however, showed deterioration through the first selection cycle, i.e., the right tail of the distribution went to the left side. In the result figures, we showed the best genotypic values among the selected genotypes via genomic prediction at each selection cycle, so that the attained genotypic values appeared to go down through the first selection cycle.

### Genetic variance in a breeding population and prediction accuracy at each selection cycle

**Fig 4:**
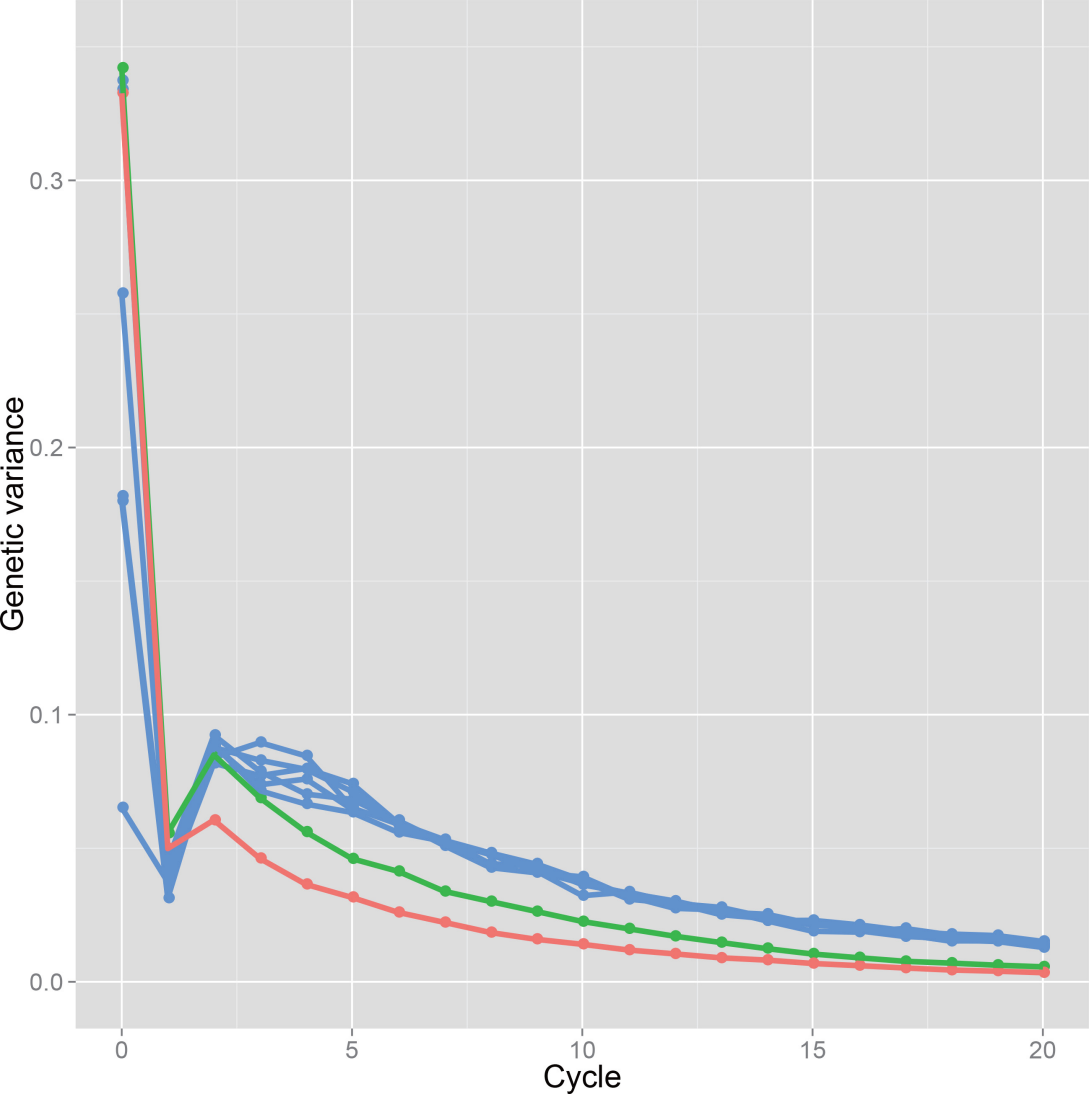
Genetic variance shown through selection cycles. Results of the discrete GS in a population derived from a single bi-parental cross of Koshihikari × Hatsushimo: red line. Results of the bulked GS: green line. Results of each subpopulation in the island model GS: blue lines.

All GS breeding schemes show similar trends in the genetic variance (Fig 4). The variance decreased after the first selection and increased somewhat after the second selection in GS breeding of all types. Then, after the third selection, the genetic variance decreased gradually. For the discrete GS and the bulked GS, both breeding strategies showed similar levels of decrease in variance at the first selection, but the level of increase was larger in the bulked GS than the discrete GS at the second selection. Because of this difference of increment of variance, the breeding population of the bulked GS maintained higher variance than that of the discrete GS in early generations. The island-model GS showed different levels of genetic variances among the initial subpopulations. At the first selection, the island-model GS showed the lower variance than the others, and increased variance more than the others. The average genotypic values of subpopulations in the island-model GS did not converge until the fifth selection cycle, although they almost converged after the sixth selection cycle (Fig 3B).

**Fig 5:**
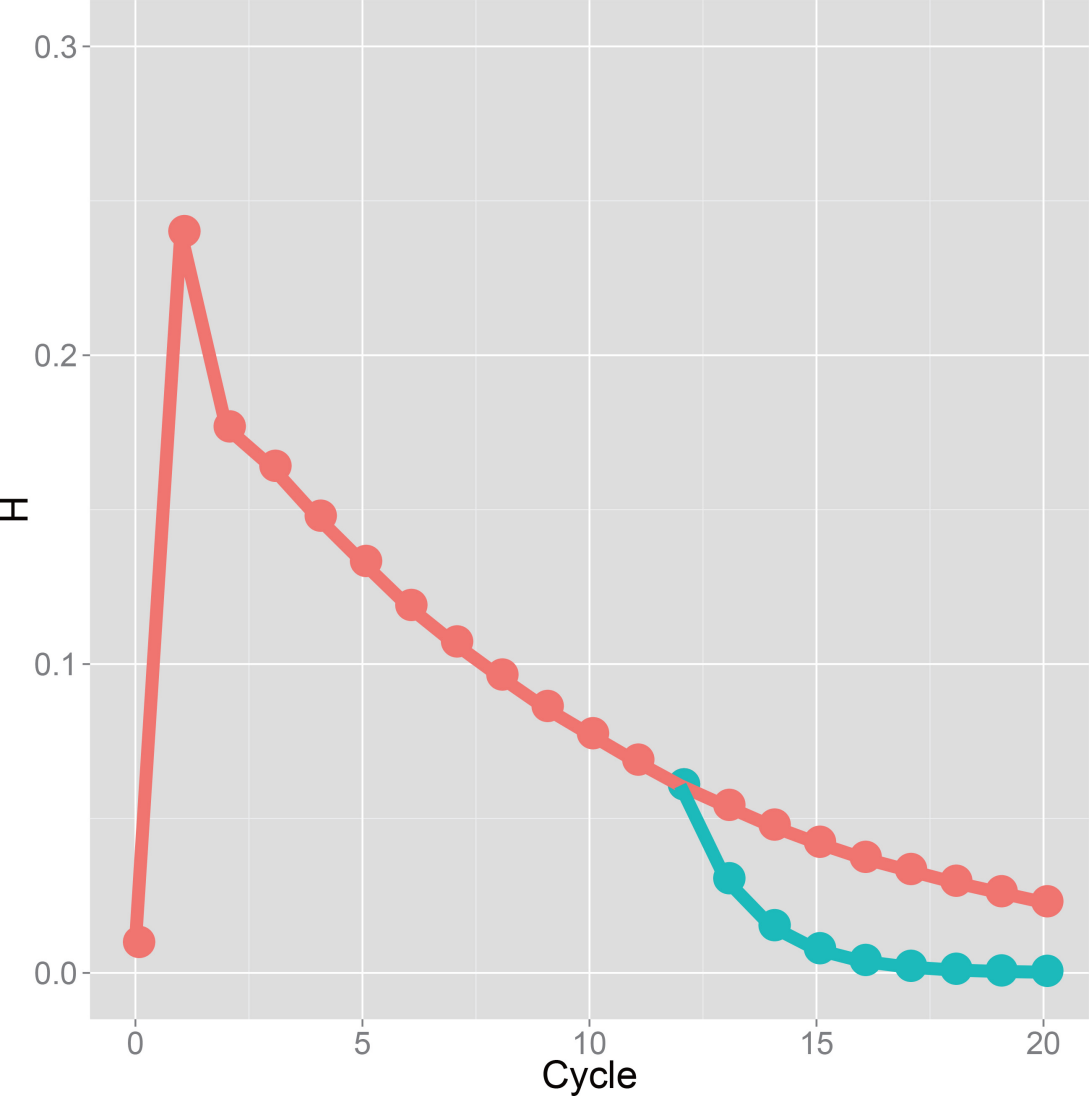
Proportion of heterozygous loci among the all SNPs in the bulked GS. Red line represents the result of the bulked GS during 20 cycles of breeding. The blue line shows results obtained from repeated inbreeding after 12 cycles of the bulked GS.

**Fig 6:**
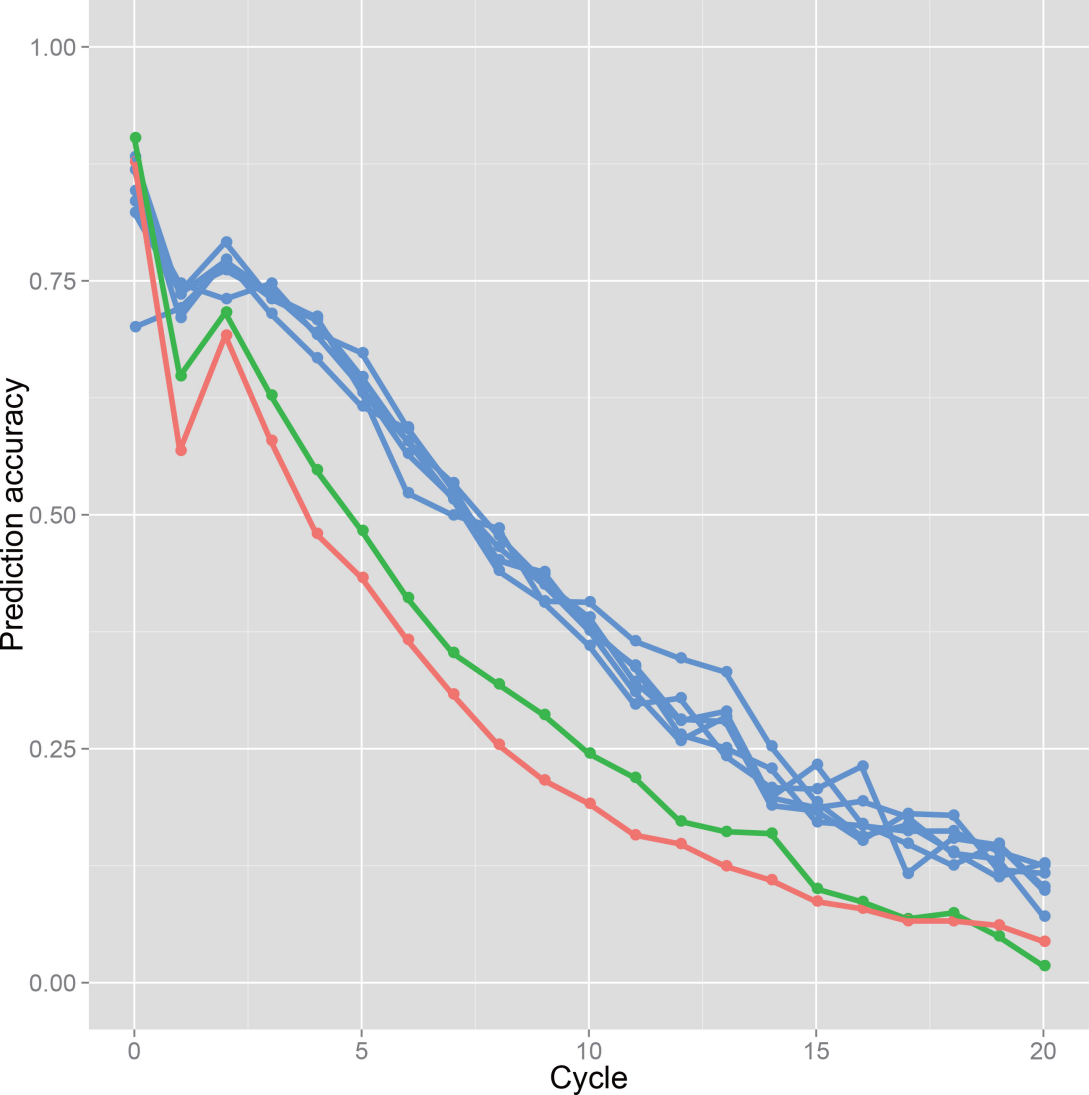
Prediction accuracy attained through selection cycles. Results of the discrete GS in a population derived from a single bi-parental cross of Koshihikari × Hatsushimo: red line. Results of the bulked GS: green line. Results of each subpopulation in the island model GS: blue lines.

In the bulked GS, almost all loci (99.03% on average) were homozygous in the initial population (Fig 5), suggesting that the process of making the initial population (i.e., repeated selfing with SSD until F_6_) created inbred lines in the present situation. In the next generation, breeding population had many heterozygous loci (23.98%). The proportion of fixed loci increased rapidly with repeated selections. Fig 6 presents the proportion of fixed loci in the bulked GS. It was 19.63% in the initial population. The proportion increased respectively to 60.48%, 75.25%, 85.90%, and 92.36% in the fifth, tenth, fifteenth, and twentieth generations. The increase of the number of fixed loci resulted in a rapid decrease of the proportion of heterozygous loci, i.e., increased number of homozygous loci (Fig 5). For the bulked GS, the homozygosity was 93.93% on average after the 12th cycle (i.e., the timing where the bulked GS has reached a plateau). Under circumstances in which inbreeding was conducted after 12 cycles of the bulked GS, the homozygosity reached 99.24%, on average, through the three cycles of inbreeding (i.e., SSD procedure).

The prediction accuracy at each selection cycle showed the same trend as that of genetic variance in the all GS breeding schemes (Fig 6). The first selection, at which the training population included the predicted candidates, attained the highest prediction accuracy. Then it decreased just after the first selection and increased slightly immediately after the second selection. After the third selection, the prediction accuracy declined gradually. The decline of accuracy immediately after the first selection was smaller in the bulked GS than in the discrete GS (Fig 6A and 6B). The island-model GS showed a much smaller decrease of accuracy immediately after the first selection than the others. The prediction accuracy varied among subpopulations over the 20 selection cycles (Fig 6C).

## Discussion

### Structure of breeding population

This study performed simulations of GS breeding in rice, which is an important autogamous cereal crop. Using a real marker data of rice varieties, the population structure existing among the rice varieties can be incorporated into the simulations. First, we compared the potential of GS breeding with the maximum potential of RILs (Fig 3A). The superiority of GS breeding was demonstrated as the high efficiency of recurrent selection, in which selection and cross are repeated, even when single-round robin was applied instead of random mating. Results suggest that recurrent selection with repeated selections and crosses can make various combinations of alleles of genes in a breeding population and can gradually fix the favorite combinations of alleles in the population through cycles of selection.

For recurrent GS, it is desirable that the initial population has large genetic variation because of its ability to contribute to genetic improvement. One can readily infer that a breeding population derived from multiple bi-parental crosses can harbor lager genetic variation in breeding than a population derived from a single superior bi-parental cross, assuming equal breeding population sizes. Comparison between these breeding populations (i.e., comparison between the discrete GS and the bulked GS) proved that a breeding population derived from mixture of multiple bi-parental crosses can attain higher genotypic values than that derived from a single bi-parental cross, even when the sizes of the two populations are equal (Fig 3B). In the discrete GS, we presented the result of a population that attained the best genetic gain among six breeding populations (the best population among all simulation trials on average and the best population in each simulation trial are shown respectively with red and blue lines in Fig 3A). Therefore, the size of the initial breeding population was 1,080 in total in the discrete GS, which was six times larger than that in the bulked GS. This result suggests the importance of admixing a large genetic diversity into one population to create new combinations of alleles of related genes. A population derived from admixture of multiple bi-parental crosses had higher genetic diversity in an initial population, and showed more moderate decline of genetic variance than a population derived from a single bi-parental cross (Fig 4), resulting in more rapid genetic improvement and slower attainment to a plateau in the bulked GS than the discrete GS (Fig 3B).

In the simulations, the decrease in genotypic values at the first cycle (Fig 3) was observed. A plant derived from the initial breeding population is expected to have the mean of genotypic values of two parental plants that were inbred (F_6_) lines selected in the initial population based on the additive model. The plant was always worse than the superior parent. The attained genotypic values (the maximum of true genotypic values) at the first cycle was lower than the initial population, although the mean of breeding population improved (e.g., S4 Fig)

### Island-model GS

In this study, we first proposed the island-model GS that was inspired by the island model in EAs, and evaluated the efficiency of the island-model GS using a computer simulation reflecting the situation of plant breeding. The island-model GS involved some uncertainty about its success because of differences between plant breeding and EAs: (i) mutations occur frequently in EAs, but they are limited in an actual plant population; (ii) EAs can simulate a large population involving wide variation, although the size of breeding population is limited in plant breeding; and (iii) EAs can gain high ability through numerous selection cycles in a short time using computer resources, although plant breeding requires much time to evaluate and select individuals. We used a population derived from multiple bi-parental crosses as an initial breeding population in the simulation of the island-model GS. In the simulation, the island-model GS showed a good performance particularly in the later cycles (Fig 3B; Table 1). The result suggests that the small population size (i.e., 180 individuals in this study) is sufficient to implement the island-model GS in actual breeding. Single-round robin was applied as a rule for making crossings because of the difficulty of random mating in autogamous species, causing no problem in implementing the island-model GS. These results suggest that the island-model GS is efficient as a selection strategy even though differences exist between actual breeding and EAs.

**Table 1:** Summary of the simulation results from three GS breeding procedures.

		Discrete GS (Koshihikari × Hatsushimo)	Bulked GS	Island-model GS
**Genotypic value**	**Cycle 5**	2.18	**2.56**	2.28
	**10**	2.40	2.96	**3.13**
	**15**	2.44	3.05	**3.46**
	**20**	2.44	3.05	**3.59**
**Selection cycles to reach the plateau**		7–8 cycles	12–13 cycles	> 20 cycles
**Proper situation**		Only two lines are available.	A new variety is required in a short time.	Long-term selection is desirable.

Genotypic values at the 5, 10, 15, and 20 cycles are shown as average. Bold letters represent the highest genotypic value at each cycle.

For GS breeding, the rapid decrease of genetic improvement becomes a daunting problem hindering long-term selection. Jannink [28] described that the reason for the decrease was the loss of favorable alleles in a breeding population. In the island-model GS, it was expected that different alleles were selected in different subpopulations so that different subpopulations involved different genetic variation. Because of the effect of the maintenance of genetic variation in a whole population, the authors expected that the island-model GS has the potential of realizing the long-term GS. In our simulations, the island-model GS attained higher genotypic values than the other GS methods did (Fig 3B). The result suggests that the island-model GS holds a clue to long-term GS.

It is important for all breeding programs to maintain genetic diversity in a breeding population. For the bulked GS, a particular family might be selected preferably at the first selection cycle because the initial breeding population is composed of multiple families derived from bi-parental crosses. Actually, in 99 out of 100 simulation trials, one or more family disappeared at the first selection cycle. For 75 out of 100 trials, the selected plants showed selection bias (chi-square tests showed significant gaps from the equal proportion for each family; *p* < 0.05). In the island-model GS, selection bias was prevented by assuming that each family derived from a single bi-parental cross as an initial subpopulation. That is, it was important to separate families, each of which was derived from a single cross, into different subpopulations. The island-model GS attained lower gain in later generations when the initial subpopulations of inbred lines were constructed randomly, irrespective of the family membership of the inbred lines than when they were constructed according to the family membership (S3 Fig). This result also suggests the importance of separating each family into an initial subpopulation. Therefore, the genetic difference among subpopulations is an important factor that makes the island-model GS beneficial compared to bulked GS. Moreover, the island-model GS reached a plateau later than the bulked GS (Fig 3B). Improvement in the bulked GS was rapid in the initial cycles (Fig 3B) because selection bias at the first cycle favored populations derived from a specific cross with high ability. This rapid improvement also led the decline of genetic variance and fast plateau of improvement in the bulked GS. In the island-model GS, different genetic variations were conserved in each subpopulation, resulting in maintenance of genetic diversity in a whole population. Migration of alleles of genes that was attained by exchanging parents (i.e., selected plants) between subpopulations contributed to the improvement of genetic potential throughout the population.

The balance between the genetic improvement of a whole population and the maintenance of genetic diversity among subpopulations can be related to migration rates. For this study, we assumed the migration size as one, which seemed a limit when the selected size was three in each subpopulation. This migration size worked efficiently in the simulations. When the migration size was two, the attained genotypic value was not much different, but the characters of subpopulations were unified earlier (S1 Fig). That is true because the large number of exchanged parents resulted in the early assimilation between subpopulations, then the same situation as when the initial subpopulations were made randomly. In the present study, even when the migration size was one, the migration rate was large (i.e., 1/3). In such a case, the migrants should have strong competitiveness in the subpopulation. Even under the high migration rate, the island-model GS with the migration interval of one (i.e., migration occurred every generation) yielded the best results among the intervals of 1–5 cycles with the same size of exchanged individuals in the simulation (S2 Fig). Results suggest that the frequent exchange of genotypes results in high efficiency of genetic improvement in the whole population, even with a high migration rate. In the simulations of the island-model GS, small population size and strong selection intensity were assumed. Selecting only three plants in one subpopulation produced a severe genetic bottleneck in the subpopulation. Because of the severe bottleneck, genetic variance in each subpopulation decreased drastically in two or three cycles of selection without migration (S5 Fig). Although the drastic decrease in genetic variation in each subpopulation resulted in the lower genetic gain through 20 selection cycles of the island-model GS with longer migration intervals, the genetic gain did not reach a plateau through the 20 cycles (S2 Fig). This result might suggest that genetic variation was maintained among subpopulations, which contributed to the utilization of large genetic variation in breeding process and the slow but long-term genetic improvement. S6 Fig shows the result of principal component analysis in 3,102 markers of a breeding population through five cycles of the island-model GS. When the migration interval was one, genotypes in different subpopulations were admixtures in several cycles. However, when the migration interval was greater than one, different subpopulation underwent selection to different direction (i.e., different subpopulation showed genotypes of different types). Wright [29] described that random differentiation tends to cause different adaptive trends and different processes of selection in different subpopulations even under uniform environmental conditions. Our simulations of the island-model GS, in which we imposed selection to the identical direction for all subpopulations, might follow this situation. The island-model GS with longer migration intervals promotes the utilization of large genetic variations in a whole population. In a breeding program, it might be better to conduct the island-model GS with the shorter migration interval because of the demand of release of new cultivars in a shorter period of time. However, if breeders can spend much time or if they can hope for long-term selection, the migration interval should be decided based on the balance between the pace of improvement and the pace of reaching a plateau.

### Suggestion for breeding of autogamous plants

The potential of the island-model GS in the breeding of an autogamous species was demonstrated via breeding simulations. For the actual plant breeding, distinctness, uniformity, and stability are necessary to release new cultivars [4]. In autogamous crop species, pure lines are made as a new cultivar to realize uniformity and stability. In general, cultivars in a market experienced from 6 to 7 cycles of selfing [4]. Considering the rapid fixation of alleles in recurrent selection shown as the rapid decline of the genetic variance (Fig 4), a few cycles of self-pollination after the genetic improvement reached a plateau sufficient to yield highly homozygous genotypes. In our simulation, only three cycles of self-pollination were sufficient to attain homozygosity higher than 99% after 12 cycles of the bulked GS (Fig 5).

The GS method presented in this paper emphasized the maintenance of genetic variation in a breeding population to select autogamous species. However, an earlier report [30] suggested the GS process with selecting more homozygous individuals. That study demonstrated that a large genetic variance is caused by self-fertilization [31], thereby improving genetic ability in GS. In their process, however, few new combinations of genes would appear in a breeding population. As suggested by the present study, GS with recurrent selection is a good strategy, especially for a long-term breeding.

Spindel et al. [32] suggested that GS might be effective for rice breeding when the target trait was controlled by a number of genes. They demonstrated that GS attained higher prediction accuracy than the conventional pedigree method and the method using a few markers with large effects for predicting grain yield, which is controlled by numerous small effect genes using 332 rice inbred lines. Their result encourages our assumption that recurrent GS can be performed with high accuracy. In our simulation study, although the markers were fewer than their recommended value (one marker every 0.2 cM), it is expected that GS can attain higher accuracy than the conventional selection methods. Spindel et al. [32] provided an example of procedures of rice breeding incorporating GS, in which GS was conducted instead of phenotypic selection during the conventional breeding procedure using inbreeding. The recurrent GS, which was proposed in the present study, might attain higher genetic gain than breeding with repeated inbreeding.

In this study, GS was conducted to evaluate a single plant accurately. However, the prediction accuracy declined with repeated selection cycles (Fig 6). The main reason for the decline is expected to be the increasing genetic distance between the training and breeding populations. In the simulation study of GS in barley (*Hordeum vulgare* L.) [28], the prediction model was updated every cycle by making doubled haploids after selection. In the present simulation, a prediction model was built based on 1,080 F_6_ lines, and was used throughout a breeding program. Selection accuracy decreased with repeated selections (Fig 6). If the prediction model can be updated, then selection accuracy would be improved (e.g., Iwata et al. 2011; Jannink 2010; Yabe et al., 2013; Yabe et al., 2014 [28, 33–35]). Updating a prediction model, however, requires great effort and time. The present results show the potential to use one prediction model, which can be built with RILs families, for a long time. The phenotype and marker genotype data of RILs or backcross inbred lines (BILs) are usually collected in public and private sectors, suggesting that breeders can use existing segregating populations and/or their marker and phenotypic data to build a prediction model. The optimal timing for updating a prediction model should be considered based on time, cost, and effort for preparing a new training population, and based on the accuracy of the updated model. Simulations with model updating were conducted to confirm the possibility that model updating increases the efficiency of genetic improvement (S7 Fig). For the simulations, we selected one realistic procedure from several possible ones. The simulations were performed for the bulked GS scenario. A prediction model was updated twice during the 20 cycles of selection using inbred lines derived from plants selected from the breeding population. Model updating increased the prediction accuracy and achieved genetic improvement only slightly. Even when the number of inbred lines used for updating the model was increased, the prediction accuracy and genetic gain did not improve to any great degree. Results suggest that low genetic variation in the breeding population impeded the genetic improvement and the increment of prediction accuracy under the model updating. For the present simulations with the model updating, we used inbred lines derived from the breeding population, in which numerous QTL had been fixed when the updated model was available (Fig 4). Based on the simulations conducted for this study, the model updating did not strongly affect the GS breeding efficiency. To improve the GS breeding efficiency, genetic variation introduced from other breeding populations would be more useful than the model updating. However, further analysis is necessary to optimize the resource allocation for model updating and the introduction of new genetic variation.

For this study, it was assumed that the initial population comprised of six families derived from six combinations of bi-parental crosses. The seven varieties used as parents of the initial breeding and training population were selected to represent the genetic diversity in the 112 cultivars well based on their marker genotypes. It is also possible to choose parental varieties based on phenotypic variation of target traits required for breeding objectives. We often conduct breeding programs under certain restrictions (e.g., a lower limit of seed or fruit quality). Therefore, it might be efficient to choose parents according to the required level of phenotypic values rather than genetic diversity of marker genotypes. In this case, however, one might miss some useful alleles harbored by inferior varieties.

For island-model GS, it is possible for multiple breeding stations work together to create a new cultivar. A breeding population has been selected according to the local adaptation and maintains different genetic diversity from other populations. We can use this situation in island-model GS by assuming a breeding population in each region as a subpopulation. In each region, breeders can conduct their breeding programs using their own population. They can occasionally exchange a part of their cultivars for cultivars of other regions to introduce new genetic variation into their population. Actually, breeding procedures of this type have been conducted consciously or unconsciously in the traditional breeding of various crop species. The island-model GS is intended to balance genetic improvement and the maintenance of genetic diversity in a more explicit manner.

## Conclusions

This report is the first of a study investigating the potential of an island model for GS breeding. This study demonstrates that recurrent GS is efficient for the breeding of autogamous crops. Recurrent GS produces novel combinations of genes in a breeding population and is expected to attain higher genetic gain than conventional breeding methods by inbreeding. For recurrent GS, it is important to involve large genetic variation, so that a greater number of novel combinations of genes can be produced. The island-model GS, which was derived from population genetics and which has shown high efficiency in EA field, is effective to improve genotypic values while maintaining genetic variation in a breeding population. In the island-model GS, subpopulations maintain different genetic variation by involving different variations and conducting selection to different directions. The island model GS improves a population slowly but for a long time, resulting in high genetic gain using genetic variation while avoiding any discarding of favorable genes. By the recurrent GS, autogamous breeding might break through the barrier of genetic improvement. Moreover, island-model GS can involve wide genetic variation in a breeding program and realize long-term selection.

## Supporting Information

S1 Fig Genotypic values attained through selection cycles in the island-model GS. Red lines represent the genotypic values attained when the migration interval was 1 and the number of exchanged individuals was 1. Blue lines show results obtained when the migration interval was 1 and the number of exchanged individuals was 2.
(PDF)

S2 Fig Genotypic values attained through selection cycles in the island-model GS. Red lines represent the genotypic values attained when the migration interval was 1 and the number of exchanged individuals was 1. Blue lines show the results of following settings: (a) the migration interval was 2 and the number of exchanged individuals was 1, (b) the migration interval was 3 and the number of exchanged individuals was 1, (c) the migration was 4 and the number of exchanged individuals was 1, and (d) the migration interval was 5 and the number of exchanged individuals was 1.
(PDF)

S3 Fig Genotypic values attained through selection cycles in the island-model GS. Red lines represent the genotypic values attained when the migration interval was 1 and the number of exchanged individuals was 1, in which the initial population was separated according to their parents. Blue lines show the results when the migration interval was 1 and the number of exchanged individuals was 1, whereas the initial population was randomly allocated to six subpopulations.
(PDF)

S4 Fig Distribution of genotypic values at two simulation trials in the bulked GS. The distributions shown in red, green, and blue respectively show the values of the initial population, the population experienced one selection cycle, and the population experienced two selection cycles.
(PDF)

S5 Fig Genetic variance attained through selection cycles in the island-model GS. Red lines represent the genotypic values attained when the migration interval was 1 and the number of exchanged individuals was 1. Blue lines show results of the following settings: (a) the migration interval was 2 and the number of exchanged individuals was 1, (b) the migration interval was 3 and the number of exchanged individuals was 1, (c) the migration interval was 4 and the number of exchanged individuals was 1, and (d) the migration interval was 5 and the number of exchanged individuals was 1.
(PDF)

S6 Fig Principal component analysis in 3,102 markers of a breeding population through five cycles in the island-model GS. x-axis and y-axis respectively show the first and second principal component. Different colors represent different subpopulations. The numbers represented at the top of the plot show the migration interval. The numbers at the right side of the plot show the selection cycles. This figure presents one simulation trial out of 100 trials.
(PDF)

S7 Fig Impact of updating a prediction model. Genotypic values (a) and prediction accuracy (b) through selection cycles in the bulked GS with model update are shown. Gray vertical lines represent the selection cycles at which the updated prediction models started be used. The green line represents the bulked GS without model updating. For updating the prediction model, 180 (or 540) lines derived from 18 plants that were selected at the 2^nd^ and 8^th^ selection cycles were used to build a new prediction model after five cycles of selfing. At the first cycle, 10 (or 30) plants were derived from one parental individual. At the subsequent four cycles, single seed decent (SSD) was adopted. The updated prediction model was available at the 8^th^ and 14^th^ selection cycles, assuming that a certain time is required for selfing and field experiments for obtaining phenotypic data. In the simulations represented by the black line, a prediction model was updated with the original training population and 180 lines derived as described above. In the simulations represented by the orange line, all procedures are the same as those represented by the red line, except that 30 plants were derived from one parental plant to produce 540 lines for training.
(PDF)

S1 Table 112 Japanese rice cultivars used in this study with pedigree. (PDF)
