# Supplementary material for: Island-Model Genomic Selection for Long-Term Genetic Improvement of Autogamous Crops
Source: PLoS One. 2016 Apr 26;11(4):e0153945. doi: 10.1371/journal.pone.0153945 (PMC4846018; doi:10.1371/journal.pone.0153945)
Supplement: S1 Fig — Red lines represent the genotypic values attained when the migration interval was 1 and the number of exchanged individuals was 1. Blue lines show results obtained when the migration interval was 1 and the number of exchanged individuals was 2. (PDF) [file pone.0153945.s001.pdf]

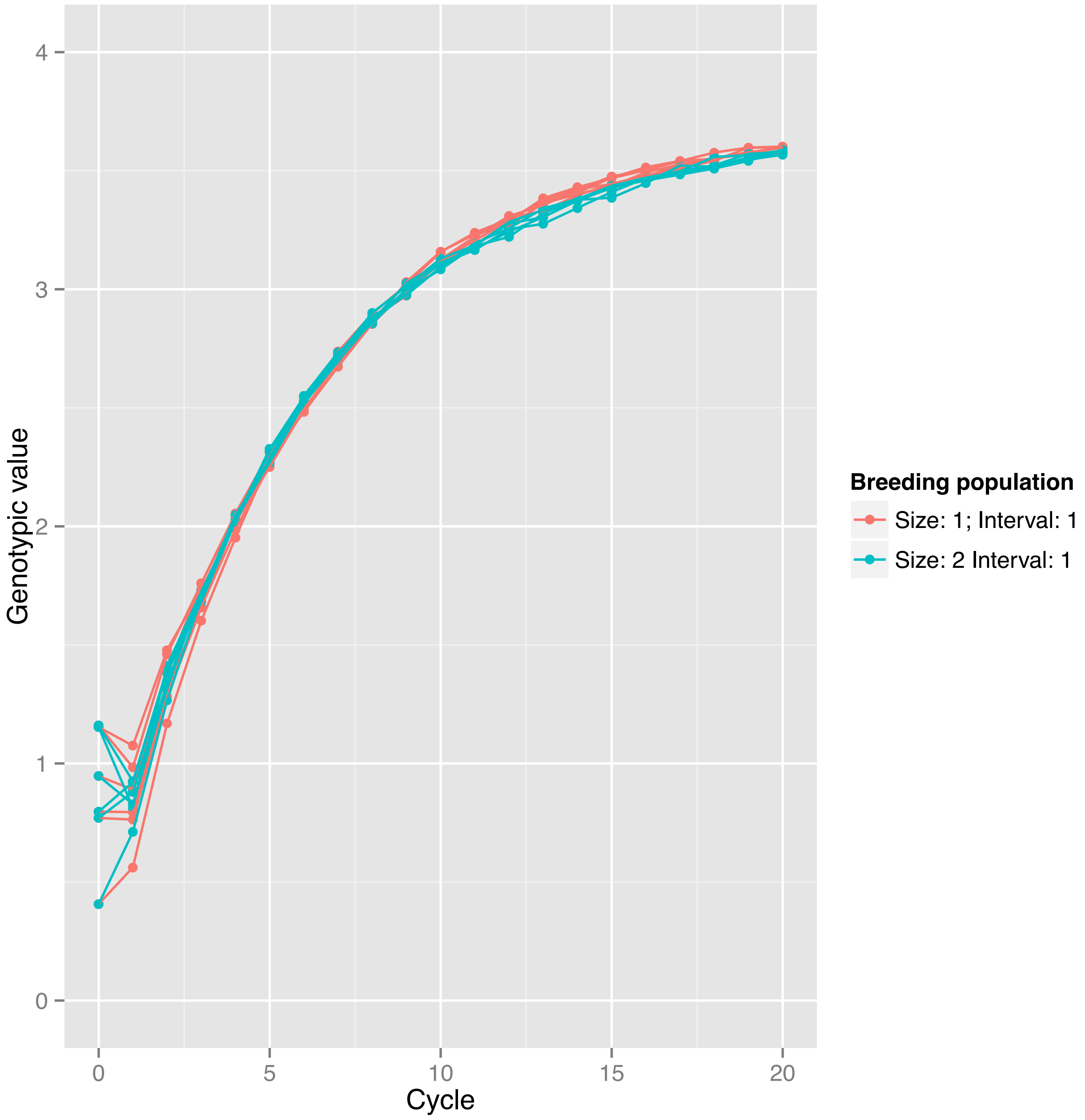

S1 Fig. Genotypic values attained through selection cycles in the island-model GS. Red lines represent the genotypic values attained when the migration interval was 1 and the number of exchanged individuals was 1. Blue lines show results obtained when the migration interval was 1 and the number of exchanged individuals was 2.
