# Supplementary material for: Island-Model Genomic Selection for Long-Term Genetic Improvement of Autogamous Crops
Source: PLoS One. 2016 Apr 26;11(4):e0153945. doi: 10.1371/journal.pone.0153945 (PMC4846018; doi:10.1371/journal.pone.0153945)
Supplement: S4 Fig — The distributions shown in red, green, and blue respectively show the values of the initial population, the population experienced one selection cycle, and the population experienced two selection cycles. (PDF) [file pone.0153945.s004.pdf]

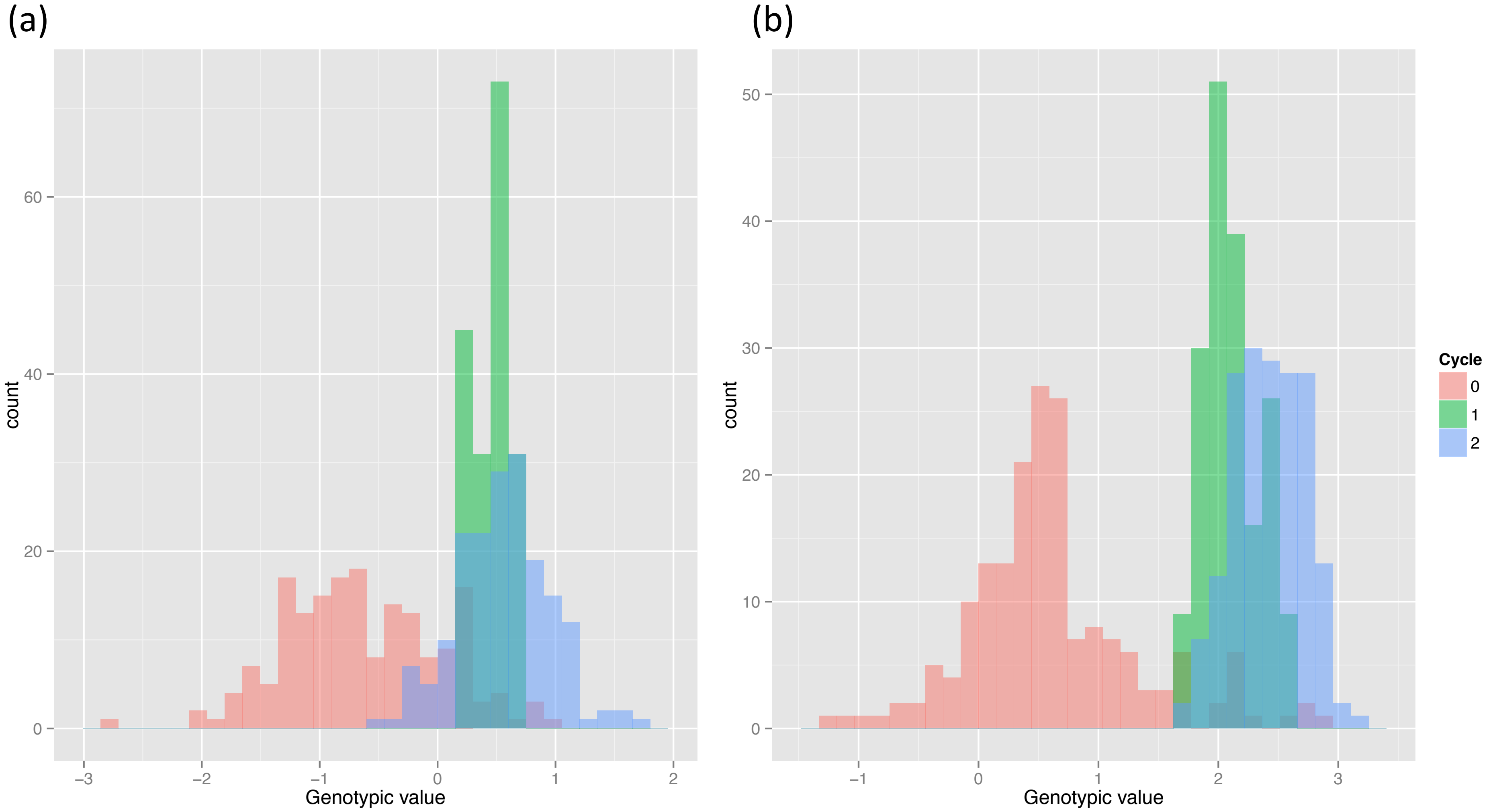

S4 Fig. Distribution of genotypic values at two simulation trials in the bulked GS. The distributions shown in red, green, and blue respectively show the values of the initial population, the population experienced one selection cycle, and the population experienced two selection cycles.
