# Supplementary material for: Island-Model Genomic Selection for Long-Term Genetic Improvement of Autogamous Crops
Source: PLoS One. 2016 Apr 26;11(4):e0153945. doi: 10.1371/journal.pone.0153945 (PMC4846018; doi:10.1371/journal.pone.0153945)
Supplement: S6 Fig — x-axis and y-axis respectively show the first and second principal component. Different colors represent different subpopulations. The numbers represented at the top of the plot show the migration interval. The numbers at the right side of the plot show the selection cycles. This figure presents one simulation trial out of 100 trials. (PDF) [file pone.0153945.s006.pdf]

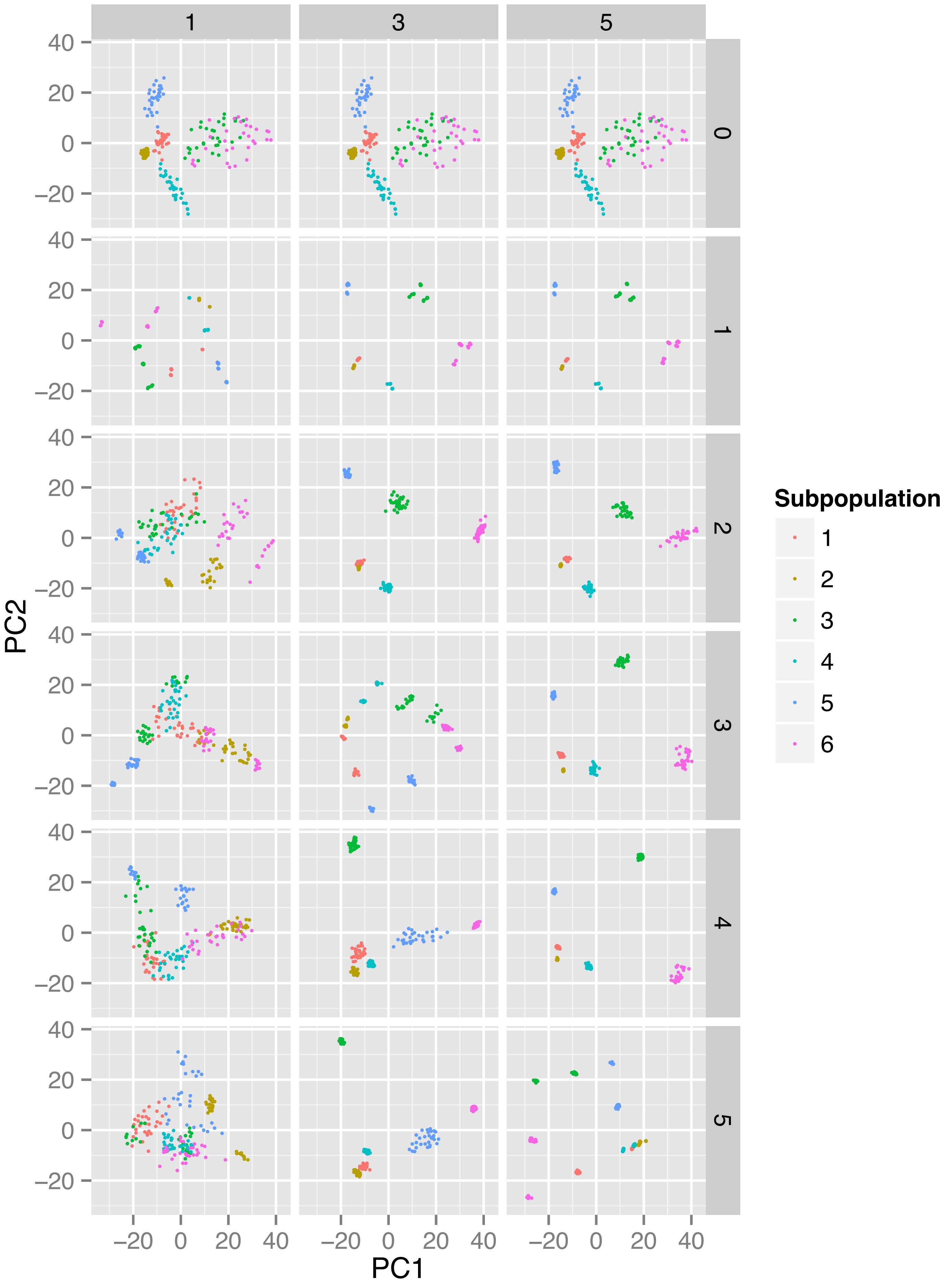

S6 Fig. Principal component analysis in 3,102 markers of a breeding population through five cycles in the island-model GS. x-axis and y-axis respectively show the first and second principal component. Different colors represent different subpopulations. The numbers represented at the top of the plot show the migration interval. The numbers at the right side of the plot show the selection cycles. This figure presents one simulation trial out of 100 trials.
