# Supplementary material for: Island-Model Genomic Selection for Long-Term Genetic Improvement of Autogamous Crops
Source: PLoS One. 2016 Apr 26;11(4):e0153945. doi: 10.1371/journal.pone.0153945 (PMC4846018; doi:10.1371/journal.pone.0153945)
Supplement: S7 Fig — Genotypic values (a) and prediction accuracy (b) through selection cycles in the bulked GS with model update are shown. Gray vertical lines represent the selection cycles at which the updated prediction models started be used. The green line represents the bulked GS without model updating. For updating the prediction model, 180 (or 540) lines derived from 18 plants that were selected at the 2nd and 8th selection cycles were used to build a new prediction model after five cycles of selfing. At the first cycle, 10 (or 30) plants were derived from one parental individual. At the subsequent four cycles, single seed decent (SSD) was adopted. The updated prediction model was available at the 8th and 14th selection cycles, assuming that a certain time is required for selfing and field experiments for obtaining phenotypic data. In the simulations represented by the black line, a prediction model was updated with the original training population and 180 lines derived as described above. In the simulations represented by the orange line, all procedures are the same as those represented by the red line, except that 30 plants were derived from one parental plant to produce 540 lines for training. (PDF) [file pone.0153945.s007.pdf]

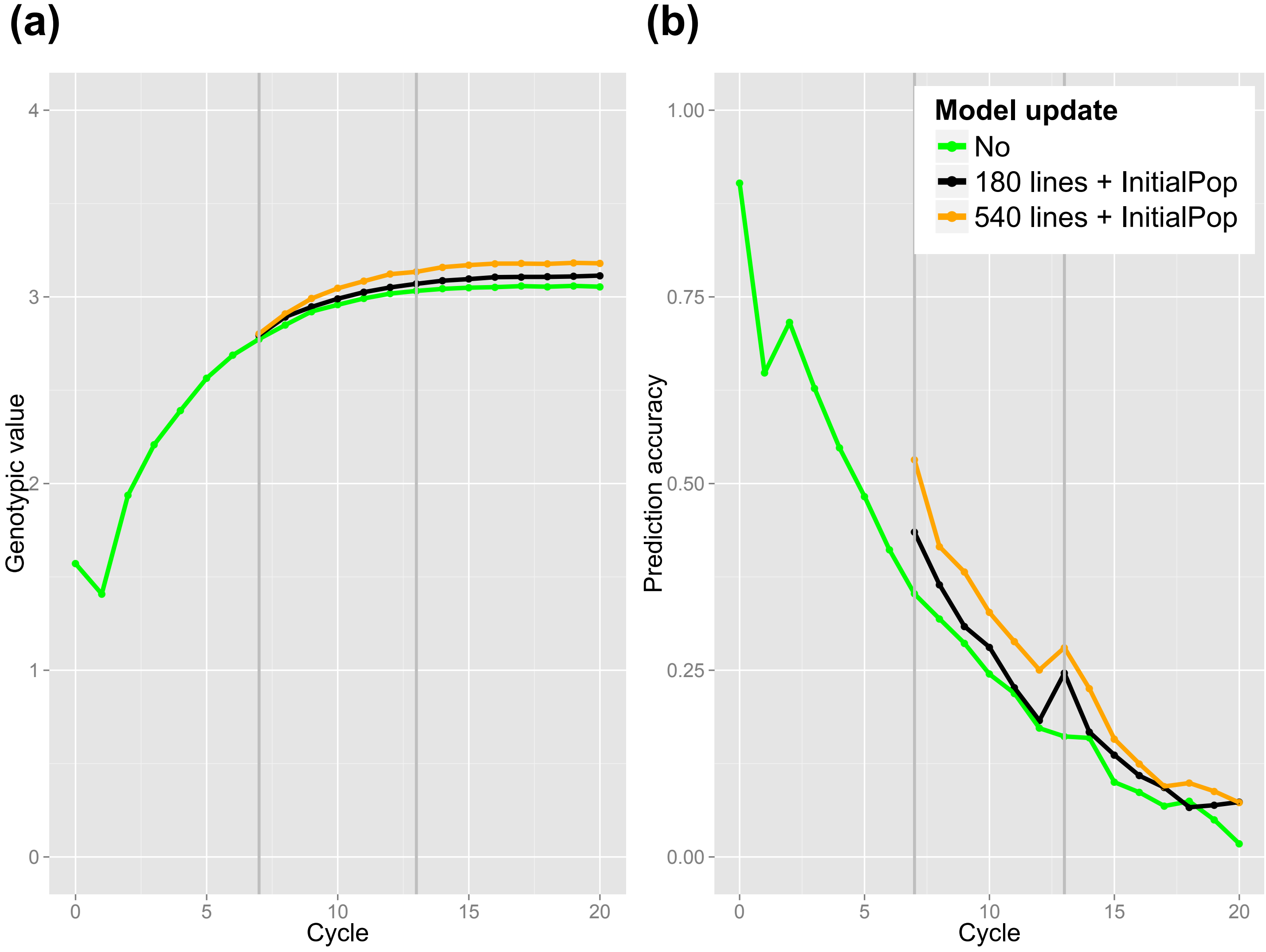

S7 Fig. Impact of updating a prediction model. Genotypic values (a) and prediction accuracy (b) through selection cycles in the bulked GS with model update are shown. Gray vertical lines represent the selection cycles at which the updated prediction models started be used. The green line represents the bulked GS without model updating. For updating the prediction model, 180 (or 540) lines derived from 18 plants that were selected at the 2nd and 8th selection cycles were used to build a new prediction model after five cycles of selfing. At the first cycle, 10 (or 30) plants were derived from one parental individual. At the subsequent four cycles, single seed decent (SSD) was adopted. The updated prediction model was available at the 8th and 14th selection cycles, assuming that a certain time is required for selfing and field experiments for obtaining phenotypic data. In the simulations represented by the black line, a prediction model was updated with the original training population and 180 lines derived as described above. In the simulations represented by the orange line, all procedures are the same as those represented by the red line, except that 30 plants were derived from one parental plant to produce 540 lines for training.
