# Supplementary material for: Island-Model Genomic Selection for Long-Term Genetic Improvement of Autogamous Crops
Source: PLoS One. 2016 Apr 26;11(4):e0153945. doi: 10.1371/journal.pone.0153945 (PMC4846018; doi:10.1371/journal.pone.0153945)
Supplement: S1 Table — (PDF) [file pone.0153945.s008.pdf]

S1 Table. 112 Japanese rice cultivars used in this study with pedigree.

No.	Variety name	Improved lines or landrace	Origin	Region	Pedigree	Registration (bred)
1	Kirara 397	Improved	Hokkaido	Hokkaido	Shimahikari/Kitaake	1990
2	Hoshinoyume	Improved	Hokkaido	Hokkaido	Akitakomachi/Dohoku 48//Kirara 397	2000
3	Yukihikari	Improved	Hokkaido	Hokkaido	Kitahikari/Tomoemasari//Kuiku 99	1986
4	Hayamasari	Improved	Hokkaido	Hokkaido	Eikei 75169/Eikei 76251	1990
5	Hatsushizuku	Improved	Hokkaido	Hokkaido	Matsumae/Jo 116//Hokkai 258	1998
6	Yukara	Improved	Hokkaido	Hokkaido	Kanto 53/Eiko	1962
7	Tsugaruroman	Improved	Aomori	Tohoku	Fukei 141/Akitakomachi	2000
8	Yumeakari	Improved	Aomori	Tohoku	Akitakomachi/Aokei 110	2002
9	Mutsuhomare	Improved	Aomori	Tohoku	Todoroki-wase/Akihikari//Fuji 329	1986
10	Fukei 175	Improved	Aomori	Tohoku	Hitomebore/Fukei 141	(1993)
11	Akihikari	Improved	Aomori	Tohoku	Toyonishiki/Reimei	1976
12	Reimei	Improved	Aomori	Tohoku	Mutant from Fujiminori irradiated by gamma-ray	1966
13	Fujisaka 5	Improved	Aomori	Tohoku	Futaba/Zengokuwase	1947
14	Toyonishiki	Improved	Miyagi	Tohoku	Sasanishiki/Ou 239	1969
15	Ouu 197	Improved	Miyagi	Tohoku	Ban 33/*Kamenoo 4*	(1937)
16	Chiyohonami	Improved	Miyagi	Tohoku	Akihomare/Koganehikari	1987
17	Hitomebore	Improved	Miyagi	Tohoku	Koshihikari/Hatsuboshi	1992
18	Manamusume	Improved	Miyagi	Tohoku	Chiyonishiki/Hitomebore	2001
19	Akitakomachi	Improved	Akita	Tohoku	Koshihikari/Ou 292	1984
20	Okiniiri	Improved	Akita	Tohoku	Chubu 47/Ou 313	1996
21	Fukuhibiki	Improved	Akita	Tohoku	Kochihibiki/Ou 316	1995
22	Himenomochi	Improved	Akita	Tohoku	Daikei 227/Koganemochi	1972
23	Kinuhikari	Improved	Niigata	Hokuriku	Shu 2800/Hokuriku 100//Nagoyutaka	1989
24	Dontokoi	Improved	Niigata	Hokuriku	Kinuhikari/Hokuriku 120	1997
25	Itadaki	Improved	Niigata	Hokuriku	Dontokoi/Shu 4695	2003
26	Norin 1	Improved	Niigata	Hokuriku	Morita-wase/Rikuu 132	1931
27	Hounen-wase	Improved	Niigata	Hokuriku	Norin 22/Norin 1	1955
28	Todoroki-wase	Improved	Niigata	Hokuriku	Shu 921/Honen-wase	1968
29	Koshihikari	Improved	Fukui	Hokuriku	Norin 22/Norin 1	1956
30	Hanaechizen	Improved	Fukui	Hokuriku	Etsunan 122/Fukuhikari	1993
31	Yukinosei	Improved	Niigata	Hokuriku	Fukou 101/Niigata 8	1990
32	Gohyakumangoku	Improved	Niigata	Hokuriku	Kikusui/Shin 200	1957
33	Koganemochi	Improved	Niigata	Hokuriku	Sinanomochi 3/Norin 17	1956
34	Koshiji-wase	Improved	Niigata	Hokuriku	Norin 22/Norin 1	1953
35	Goropikari	Improved	Gunma	Kanto-Tokai	Tsukinohikari/Koshihikari	1994
36	Fusaotome	Improved	Chiba	Kanto-Tokai	Hitomebore/Hanaechizen	1999
37	Hatsushimo	Improved	Aichi	Kanto-Tokai	Higashiyama 24/Norin 8	1950
38	Aichinokaori	Improved	Aichi	Kanto-Tokai	Hatsushimo/Mineasahi	1988
39	Matsuribare	Improved	Aichi	Kanto-Tokai	Aichi 56A/Mineasahi	1995
40	Asanohikari	Improved	Aichi	Kanto-Tokai	Koganebare//Aoisora/Hokuriku 103	1988
41	Tsukinohikari	Improved	Aichi	Kanto-Tokai	Koganebare//Aoisora/Hokuriku 103	1986
42	Daichinokaze	Improved	Aichi	Kanto-Tokai	Matsuribare/////Aoinokaze////Tsukinohikari///Aichi 77//Aichi 80/Akanezora	2002
43	Mineasahi	Improved	Aichi	Kanto-Tokai	Kanto 79/Kihou	1981
44	Norin 29	Improved	Gifu	Kanto-Tokai	Norin 8/Norin 6	1945
45	Akebono	Improved	Mie	Kanto-Tokai	Norin 12/*Asahi*	1953
46	Millenishiki	Improved	Ibaraki	Kanto-Tokai	Hinohikari/Inakei 517	2003
47	Satojiman	Improved	Ibaraki	Kanto-Tokai	Kanto 175/Etsunan 154	2005
48	Otomemochi	Improved	Akita	Tohoku	Koganemochi/Yamasenishiki	1966
49	Yamabiko	Improved	Mie	Kanto-Tokai	Chukyoasahi/Norin 22	1958
50	Nakate-shinsenbon	Improved	Aichi	Kanto-Tokai	Norin 22/Hayabusa	1950
51	Kinmaze	Improved	Aichi	Kanto-Tokai	Ryosaku/Aichinakateasahi	1948
52	Yamadanishiki	Improved	Hyogo	Kinki-Chugoku	Yamadaho/Tankanwataribune	1936
53	Norin 22	Improved	Hyogo	Kinki-Chugoku	Norin 8/Norin 6	1943
54	*Asahi*(朝日)	*Landrace*	Unknown	Unknown		Unknown
55	Reiho	Improved	Fukuoka	Kyushu	Houyoku/Ayanishiki	1969
56	Hiyokumochi	Improved	Fukuoka	Kyushu	Houyoku/Iwaimochi	1971
57	Houyoku	Improved	Fukuoka	Kyushu	*Jukkoku*/Zensho 26	1961
58	*Jukkoku*	*Landrace*	Unknown	Unknown		Unknown
59	Norin 18	Improved	Kumamoto	Kyushu	Oitamii 120/Takara	1941
60	Hinohikari	Improved	Miyazaki	Kyushu	Koganebare/Koshihikari	1989
61	Nishihomare	Improved	Miyazaki	Kyushu	Toyotama/Chugoku 45	1979
62	Koganemasari	Improved	Miyazaki	Kyushu	Nipponbare/Koganenishiki	1976
63	Yumetsukushi	Improved	Fukuoka	Kyushu	Kinuhikari/Koshihikari	1995
64	*Aikoku*	*Landrace*	Unknown	Unknown		Unknown
65	*Asahi*(旭)	*Landrace*	Unknown	Unknown		Unknown
66	*Ohba*	*Landrace*	Unknown	Unknown		Unknown
67	*Kameji*	*Landrace*	Unknown	Unknown		Unknown
68	*Kamenoo*	*Landrace*	Unknown	Unknown		Unknown
69	*Shinriki*	*Landrace*	Unknown	Unknown		Unknown
70	*Takenari*	*Landrace*	Unknown	Unknown		Unknown
71	Futaba	Improved	Aichi	Kanto-Tokai	Shinju 2/Takaneasahi	1940
72	Nipponbare	Improved	Aichi	Kanto-Tokai	Yamabiko/Sachikaze	1963
73	Nihonmasari	Improved	Saitama	Kanto-Tokai	Kochikaze/Nipponbare	1973
74	Kihou	Improved	Aichi	Kanto-Tokai	GinfujiB/Akibare	1968
75	Koganebare	Improved	Aichi	Kanto-Tokai	Nipponbare/Kihou	1981
76	Yukimaru	Improved	Hokkaido	Hokkaido	Kirara 397/Kuiku 125	1995
77	Nanatsuboshi	Improved	Hokkaido	Hokkaido	Hitomebore/Kukei 90242A//Akiho	2004
78	Taichung 65	Improved	Kyushu U.	(Taiwan)	*Kameji*/*Shinriki*	1927
79	Eiko	Improved	Hokkaido	Hokkaido	Tsurukame/Wasefukoku	1939
80	*Akage*	*Landrace*	Unknown	Unknown		Unknown
81	Fujihikari	Improved	Hiroshima	Kinki-Chugoku	R151/F_4(Fukei 71/Fukei 67//Koshihikari///Koshihikari)	1977
82	Benisengoku	Improved	Fukuoka	Kyushu	Tokaiasahi/Norin 26	1953
83	Yumehitachi	Improved	Ibaraki	Kanto-Tokai	Chiyonishiki/Kinuhikari	2000
84	Asahinoyume	Improved	Aichi	Kanto-Tokai	Aichinokaori//Tskinohikari/Aichi 65	2000
85	Akanezora	Improved	Aichi	Kanto-Tokai	Tsukinohikari/Koshihikari	1993
86	Hatsuboshi	Improved	Aichi	Kanto-Tokai	Koshihikari/Kihou	1977
87	Chiyonishiki	Improved	Aichi	Kanto-Tokai	Hatsuboshi/Toyonishiki	1986
88	Menkoina	Improved	Akita	Tohoku	Hitomebore/Akita 39	2001
89	Notohikari	Improved	Ishikawa	Hokuriku	Fukuhikari/Koshiji-wase	1986
90	Hohohonoho	Improved	Ishikawa	Hokuriku	Notohikari/Akitakomachi	(1993)
91	Yumehikari	Improved	Fukuoka	Kyushu	Koshihikari//F_2(Nishihikari/Koshihikari)	1992
92	Natsuhikari	Improved	Kagoshima	Kyushu	Katsurawase/Seinan 45	1984
93	Haenuki	Improved	Yamagata	Tohoku	Shonai 29/Akitakomachi	1993
94	Domannaka	Improved	Yamagata	Tohoku	Ibukiwase/Shonai 29	1993
95	Haruru	Improved	Yamaguchi	Kinki-Chugoku	Yamahoushi/Koshihikari	2001
96	Norin 6	Improved	Hyogo	Kinki-Chugoku	*Joshu*/*Senichi*	1936
97	Norin 8	Improved	Hyogo	Kinki-Chugoku	*Ginbozu*/*Asahi*	1937
98	*Morita-wase*	*Landrace*	Unknown	Unknown		1913
99	Rikuu 132	Improved	Akita	Tohoku	*Rikuu 20*/*Kamenoo 4*	1921
100	*Joshu*	*Landrace*	Unknown	Unknown		Unknown
101	*Senichi*	*Landrace*	Unknown	Unknown		Unknown
102	*Ginbozu*	*Landrace*	Unknown	Unknown	A variety form *Aikoku*	Unknown
103	*Rikuu 20*	*Landrace*	Akita	Tohoku	Pure line selection from *Aikoku*	1915
104	*Kamenoo 4*	*Landrace*	Aomori	Tohoku	Selection form *Kamenoo*	1915
105	*Omachi*	*Landrace*	Unknown	Unknown		Unknown
106	*Sekitori*	*Landrace*	Unknown	Unknown		Unknown
107	*Bozu*	*Landrace*	Unknown	Unknown		Unknown
108	*Shirosenbon*	*Landrace*	Unknown	Unknown		Unknown
109	Hatsunishiki	Improved	Akita	Tohoku	Norin 22/Norin 1	1954
110	Yamasenishiki	Improved	Akita	Tohoku	Norin 22/Norin 1	1962
111	Sasashigure	Improved	Miyagi	Tohoku	Norin 8/Tohoku 24	1952
112	Sasanishiki	Improved	Miyagi	Tohoku	Hatsunishiki/Sasashigure	1963
